# Emergency Position Recovery Using Forward Kinematics in Robotic Patient Positioning Systems for Radiosurgery

**DOI:** 10.3390/s25041202

**Published:** 2025-02-16

**Authors:** Alaa Saadah, Laszlo Fadgyas, Donald Medlin, Jad Saud, Jason Henderson, Tibor Koroknai, Mate Koroknai, David Takacs, Peter Panko, Xiaoran Zheng, Endre Takacs, Géza Husi

**Affiliations:** 1 Doctoral School of Informatics, University of Debrecen, Kassai útca 26, 4028 Debrecen, Hungary; 2 Medical Beam Laboratories, 108 Woodbriar Ct., Greenville, SC 29617, USA; 3 Spinotron Kft, Bethlen u. 11-17, 4028 Debrecen, Hungary; jadsaud@spinotron.hu; 4 Quiverent LLC, 2801 Wade Hampton Blvd. Ste. 115-301, Taylors, SC 29687, USA; 5 Medikai Innováció Kft., Bethlen u. 11-17, 4028 Debrecen, Hungarydavidetkcs@gmail.com (D.T.); prike18@gmail.com (P.P.); 6 Department of Physics and Astronomy, Clemson University, Clemson, SC 29631, USA; etakacs@clemson.edu; 7 Department of Electrical Engineering and Mechatronics, University of Debrecen, Ótemet˝o útca 2-4, 4028 Debrecen, Hungary; husigeza@eng.unideb.hu

**Keywords:** robotic patient positioning, radiosurgery, forward kinematics (FK), primary encoders, secondary encoders, dual-loop control, emergency interruption handling, radiation therapy, medical robotics, precision positioning

## Abstract

Precise patient positioning is paramount in radiosurgery to ensure the accurate targeting of tumors while minimizing damage to surrounding healthy tissues. This study focuses on the development and validation of a robust forward kinematics (FK) model for a robotic patient positioning system, specifically designed to handle emergency interruptions such as power loss or emergency stops. The FK model integrates data from high-resolution primary encoders within the actuators and secondary encoders post-load on each axis, providing real-time feedback and ensuring sub-millimeter positional accuracy. The control architecture leverages a dual-loop feedback system to enhance precision and stability during operation and recovery. The simulation and experimental results demonstrate that the FK model, combined with encoder feedback, reliably determines the patient bed’s exact position during interruptions and guides the system’s safe and accurate resumption of treatment. These findings underscore the critical role of FK and encoder integration in improving the safety, reliability, and precision of robotic radiosurgery systems, addressing key challenges in medical robotics for radiation therapy.

## 1. Introduction

Radiosurgery, particularly in the treatment of brain tumors, has undergone significant evolution, characterized by remarkable advancements in precision and safety. A pivotal moment occurred in the 1960s with the introduction of the Gamma Knife, a non-invasive technique that provided a groundbreaking alternative to traditional neurosurgery. By delivering highly focused radiation with exceptional accuracy, it became especially effective for treating tumors near sensitive brain structures, where surgical techniques pose significant risks [1].

Stereotactic radiosurgery (SRS) is now a cornerstone for intracranial tumor treatment, offering a single-dose, highly precise approach that minimizes damage to surrounding healthy tissue. It is widely used to treat brain metastases, with studies demonstrating its effectiveness as comparable to or better than whole-brain radiation therapy (WBRT) in certain cases. Moreover, SRS alone significantly reduces the risk of memory and cognitive side effects often associated with WBRT [2]. Advanced technologies like the Gamma Knife, CyberKnife, and stereotactic linear accelerators have further refined the accuracy and safety of SRS, making it an indispensable tool in modern neurosurgery [3].

The effectiveness of the Gamma Knife lies in its ability to minimize collateral damage to surrounding healthy tissues, a critical concern in neurosurgery, thereby significantly improving patient outcomes. Over the years, enhancements in imaging techniques—such as higher-resolution MRI and CT scans—and sophisticated treatment planning software have further increased the precision of the Gamma Knife. These advancements have enabled more complex and accurate treatment strategies for previously inoperable brain tumors [4]. However, challenges such as optimizing treatment for dynamic anatomical changes during radiation delivery remain a focus of ongoing research [5].

The origins of radiosurgery can be traced back to 1949, when Lars Leksell, a neurosurgeon at Karolinska University in Stockholm, and Bjorn Larsson, a radiobiologist, pioneered the first procedure. Their initial experiments used cyclotron-derived proton radiation, but they soon adopted cobalt-60 as a radiation source due to its effectiveness and simplicity [6]. By 1968, the Gamma Knife, exclusively designed for intracranial radiosurgery, was completed. The device utilized cobalt-60 isotopes arranged radially to deliver highly focused gamma radiation, significantly reducing the radiation load on surrounding healthy tissues [7]. This foundational design remains largely unchanged, evolving to incorporate advanced imaging and treatment planning systems.

A key aspect of Gamma Knife radiosurgery is the use of a rigid stereotactic frame that ensures precise targeting by immobilizing the patient’s head. This approach, combined with three-dimensional (3D) imaging modalities such as CT and MRI, has further enhanced treatment accuracy. These technological advancements have cemented the Gamma Knife as the “gold standard” for intracranial radiosurgery. While other technologies like linear accelerators (LINAC) and CyberKnife systems have emerged, the Gamma Knife remains uniquely suited for the high-precision treatment of brain tumors [8]. LINAC-based systems, for example, allow for broader applications, including body-wide radiosurgery, and incorporate frameless and robotic technologies. However, for intracranial tumors, the Gamma Knife’s unparalleled precision and accumulated clinical experience continue to set it apart [9].

Parallel to these developments in radiosurgery, the field of robotic-assisted surgery has undergone transformative changes. The introduction of robotic systems such as the da Vinci Surgical System in 2000 revolutionized minimally invasive surgery by setting new standards for precision and control [10]. The principles of robotic precision, which have been widely adopted in various surgical disciplines, have gradually extended to radiosurgery as well. These innovations have influenced both the design and functionality of modern radiosurgical systems, providing enhanced capabilities in terms of accuracy and reproducibility [11].

Robotic technologies have had a profound impact on radiosurgery beyond operational precision. One of the most significant contributions has been in improving patient safety and comfort by enhancing the accuracy of tumor targeting and reducing the duration of radiation exposure. Modern radiosurgical systems now employ dual-loop control mechanisms, integrating primary and secondary optical encoders, to ensure real-time feedback and adjustments during treatment [12]. This integration is crucial for maintaining alignment and positioning accuracy, especially in dynamic clinical environments where unexpected interruptions, such as patient movement or equipment malfunction, may occur [13].

The use of dual-encoder configurations in patient positioning systems (PPS) is an exemplary advancement, offering high-resolution feedback on motor positioning and movement. This real-time monitoring is essential for the accurate delivery of gamma beams, where even minor misalignments can lead to suboptimal treatment outcomes [14]. Such systems leverage real-time encoder data and forward kinematics to continuously adjust the patient’s position during radiation delivery, ensuring precise alignment even during long and complex procedures [15].

Moreover, these advanced systems are designed to respond effectively to unplanned interruptions such as power outages or emergency stops. The ability to know the exact position of each motor allows the system to resume treatment accurately from the point of interruption or safely return to a home position without compromising the patient’s treatment outcome [16]. This reliability is critical in radiosurgery, where the margin for error is minimal. Nevertheless, several challenges remain in the optimization of robotic radiosurgery, including refining real-time feedback algorithms, improving the integration of imaging technologies, and exploring ways to minimize treatment time while maximizing precision.

Our study involved a thorough review of existing research on PPS, revealing the pivotal role of advanced control systems in achieving high accuracy and reliability. For instance, a study by Johnson and Patel (2022) highlights that a PPS with dual control loops is more accurate and dependable than those with a single loop [17], a critical consideration for ensuring patient safety and precise treatment. Another research piece by Nguyen and Garcia (2021) underscores the importance of robust safety features in PPS, which help to reduce risks and improve patient outcomes [18].

In addition to ongoing research, major companies such as Siemens and Samsung are actively competing to deliver superior products [19,20]. Two of the most competitive PPS products currently available include the 6-DoF Robotic Couch System by gKteso GmbH—boasting an absolute accuracy of 0.5 mm—and the Hexapods 6-Axis Patient Positioning Couches for Radiotherapy by PI (Physik Instrumente), known for their precision but limited travel range [21].

Building on these insights, we have developed our own robotic patient positioning system (RPPS) that surpasses existing offerings. Our prototype achieves an absolute accuracy of up to 0.1 mm and offers a wider travel range, positioning it as a unique and advanced solution in the field of PPS. These improvements are clinically significant: a more extensive travel range eases the setup for complex treatment angles, while heightened accuracy reduces the risk of suboptimal targeting.

A key differentiator of the PPS is its forward kinematics-based approach for emergency position recovery. In the event of unexpected power loss or an emergency stop, the robotic control algorithm can identify the exact configuration of each joint based on encoder readings, thus enabling rapid and precise restoration of the patient’s treatment position. This high level of precision, flexibility, and resiliency is intended to address existing limitations in patient positioning, thereby improving treatment safety, comfort, and efficiency. Furthermore, while our current focus is on intracranial procedures, the modular design of the RPPS could be adapted for broader, body-wide applications by refining its software and hardware configurations to suit different anatomical targets.

This paper is structured as follows to provide a comprehensive understanding of the topic:

1. System Structure and Collaborative Mechanism: This section provides a detailed description of the PPS, highlighting its three primary subsystems—Linear Rail System, Linkage System, and Tabletop Assembly—and their integration with control components to achieve precise patient positioning.
2. MathematicalModeling of Forward Kinematics: Detailed mathematical equations used to calculate the forward kinematics based on encoder feedback, including how these models aid in emergency position recovery.
3. Materials and Methods: Technical specifications and configuration of the patient positioning system, highlighting hardware components responsible for sub-millimetric accuracy and extended travel range.
4. System Integration and Testing: The implementation of the kinematic models into the control system and subsequent testing processes, demonstrating how the system handles emergencies and power interruptions.
5. Results and Discussion: An analysis of the system’s performance, focusing on accuracy, reliability, and recovery time across various operational conditions.
6. Conclusion and Future Work: The summarization of findings and potential directions for enhancing precision, emergency recovery, and body-wide treatment capabilities in robotic patient positioning systems.

By comparing our approach with existing technologies and emphasizing the advantages of our forward kinematics-driven emergency position recovery, we underscore the innovation and clinical significance of a system capable of sub-millimetric precision. The ability to maintain this accuracy through unexpected events is paramount in ensuring that the therapeutic outcome is not compromised, highlighting how this work contributes to the broader advancement of robotic radiosurgery for both intracranial and potentially full-body treatments.

## 2. System Structure and Collaborative Mechanism

**Figure 1:**
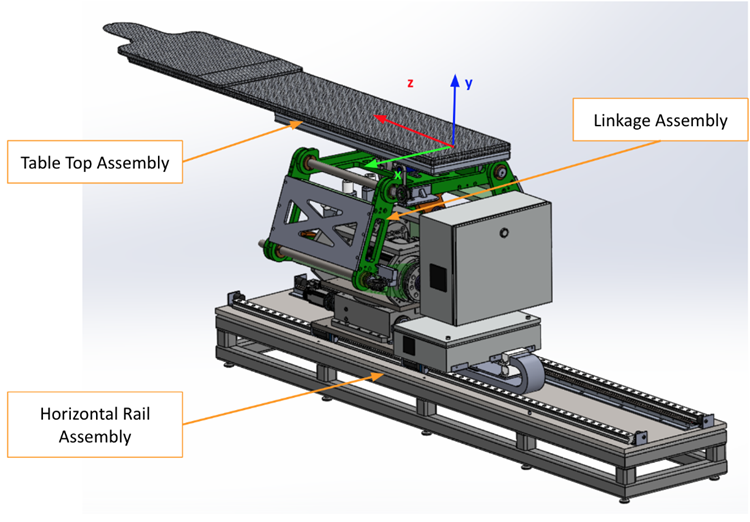
The primary PPS subsystems: Linear Rail System, Linkage System, and Table Assembly.

As shown in Figure 1, the robotic patient positioning system (PPS) is a six-degree-of-freedom (DOF) robotic platform for radiosurgery. It is designed to achieve precise patient alignment by integrating three primary mechanical subsystems with real-time control capabilities:

- Linear Rail System: Manages horizontal movement and coarse rotation, enabling the patient to be moved into or out of the treatment area.
- Linkage System: Provides vertical and angular adjustments, raising or tilting the table to align the patient’s target region with the treatment beam.
- Tabletop Assembly: Facilitates fine pitch corrections, often using a servo-driven cam mechanism, ensuring sub-millimetric orientation for accurate targeting.

These mechanical subsystems work in conjunction with a high-performance *motion controller* and servo drivers, which issue commands to servo motors (**M1–M6**) on each axis. Feedback loops from *primary encoders* (**PE**) and *secondary encoders* (**SE**) ensure real-time tracking and dynamic corrections. This dual-loop architecture compensates for any mechanical compliance or backlash, maintaining precision across all axes.

**Figure 2:**
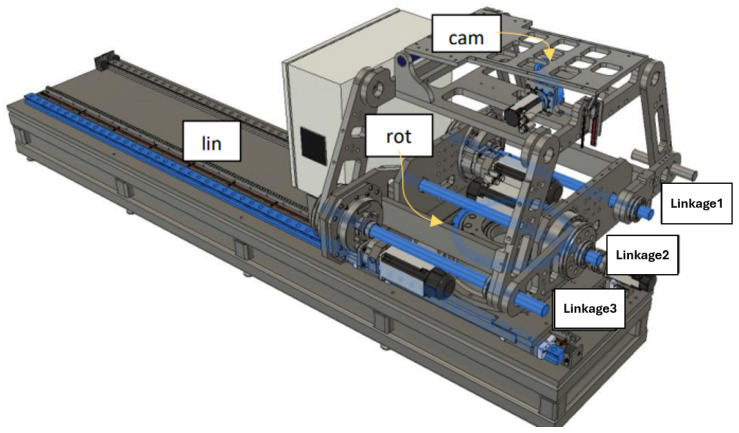
Mechanical components and corresponding movements within the PPS, including the Linear Rail System, Linkage System, and Table Assembly.

The collaborative mechanism of the PPS can be better understood by examining the roles of its components and their responsibilities for specific movements, as shown in Figure 2.

The highlighted blue sections in Figure 2 represent the key components responsible for specific movements:

- Linear Movement (Lin): The Linear Rail System is responsible for horizontal translational movement, enabling coarse adjustments to position the patient into or out of the treatment area.
- Rotational Movement (Rot): The rotary table allows for coarse rotational adjustments, helping align the patient’s target zone with the radiation beam.
- Pitching Movements (Cam): The pitching cam system provides fine angular adjustments for tilting the Tabletop Assembly, ensuring precise alignment of the treatment target.
- Vertical and Angular Movements (Linkages): The three linkage arms (Linkage1, Linkage2, Linkage3) are responsible for vertical and angular adjustments. They collaboratively raise or lower the table and perform tilting motions to align the patient accurately.

**Figure 3:**
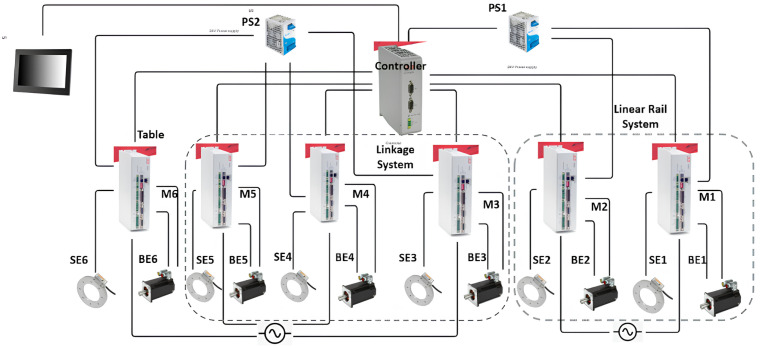
Control diagram of the PPS, showcasing the connections between motors (M), primary encoders (PE), secondary encoders (SE), and drives across the three subsystems.

The integration of the control system enables these movements with high precision. Figure 3 illustrates the detailed control mechanism.

The control system operates as follows:

- Each subsystem is controlled by a dedicated servo motor (M1–M6), with feedback from both primary (PE1–PE6) and secondary encoders (SE1–SE6).
- A central motion controller coordinates the movement of all components, issuing real-time commands and processing encoder feedback to ensure precise alignment.
- Power supplies (PS1, PS2) provide stable 24V DC power, ensuring uninterrupted operation of the system.

This integration of mechanical and control subsystems ensures that the RPPS achieves the necessary precision and reliability required for safe and effective radiosurgery.

## 3. Forward Kinematics

Forward kinematics, a foundational concept in robotics and mechanical systems, revolves around the determination of the end effector’s position and orientation based on given joint parameters and link lengths. Unlike its counterpart, inverse kinematics, which seeks to find joint parameters given a desired end effector position, forward kinematics is a direct mapping from the joint space to the workspace [22]. This computational model becomes essential in scenarios where it is necessary to predict the movement outcome based on joint values [23].

### Denavit–Hartenberg (DH)

**Figure 4:**
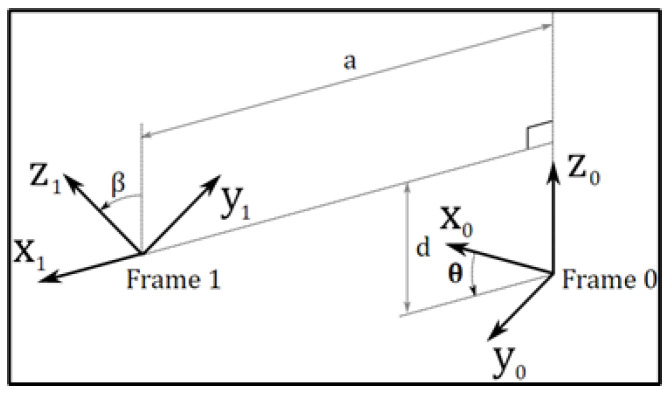
Denavit–Hartenberg (DH).

One of the most important methods to manipulate a robot based on the kinematics modeling is Denavit–Hartenberg (DH); it is a prominent approach in kinematic modeling for robot manipulation and plays a vital role in achieving effective control over robotic systems [24]. This method enables the traversal from the base frame to the end effector frame (in our study target point) by sequentially transitioning through intermediate frames, irrespective of the robot’s dynamics or specific characteristics. It delineates the transformations required, encompassing both the rotational and translational motions of the manipulator, as illustrated in Figure 4, in order to obtain the transformation matrix.

Starting from that, we can generate the (3) matrix of our robot after determining the assignment of the manipulator frames—which is defined in Figure 4 as [23]:

(1) $$T_{i}=\mathrm{Rot}\left(z,\theta_{i}\right)\mathrm{Trans}\left(d_{i}\right)\mathrm{Trans}\left(a_{i}\right)\mathrm{Rot}\left(x,\alpha_{i}\right)$$

*T_i_*, the final matrix, will be the result of multiple of those matrices

(2) $$T_{i}=\left[\begin{array}{cccc} c\theta_{i} & -s\theta_{i} & 0 & 0\\ s\theta_{i} & c\theta_{i} & 0 & 0\\ 0 & 0 & 1 & d_{i}\\ 0 & 0 & 0 & 1 \end{array}\right]\left[\begin{array}{cccc} 1 & 0 & 0 & a_{i}\\ 0 & c\alpha_{i} & -s\alpha_{i} & 0\\ 0 & s\alpha_{i} & c\alpha_{i} & 0\\ 0 & 0 & 0 & 1 \end{array}\right]$$

This equation can be translated into matrices, and by multiplying them, we obtain the final *T_i_* as follows:

(3) $$T_{i}=\left[\begin{array}{cccc} \cos\theta_{i} & -\cos\alpha_{i}\sin\theta_{i} & \sin\alpha_{i}\sin\theta_{i} & a_{i}\cos\theta_{i}\\ \sin\theta_{i} & \cos\alpha_{i}\cos\theta_{i} & -\sin\alpha_{i}\cos\theta_{i} & a_{i}\sin\theta_{i}\\ 0 & \sin\alpha_{i} & \cos\alpha_{i} & d_{i}\\ 0 & 0 & 0 & 1 \end{array}\right]$$

where

- $a_{i}$: The distance between the $z_{i}$ and $z_{i+1}$ axes along the $x_{i}$ axis.
- $\alpha_{i}$: The angle between the $z_{i}$ and $z_{i+1}$ axes along the $x_{i}$ axis.
- $d_{i}$: The distance between the $x_{i}$ and $x_{i+1}$ axes along the $z_{i}$ axis.
- $\theta_{i}$: The angle between the $x_{i}$ and $x_{i+1}$ axes along the $z_{i}$ axis.

## 4. PPS Subsystems Functionality Description and Coordinate Frame Assignment

This vital system plays a crucial role in facilitating precise patient alignment in a variety of medical scenarios. Its construction and functionality embody several distinctive features that contribute to its effective performance. These characteristics span across the system’s three main components: the Linear Rail System, the Linkage System, and the Table Assembly. Each component introduces unique attributes that, when combined, result in a highly efficient, versatile, and patient-friendly positioning system.

### 4.1. Frame Assignments for the PPS

**Figure 5:**
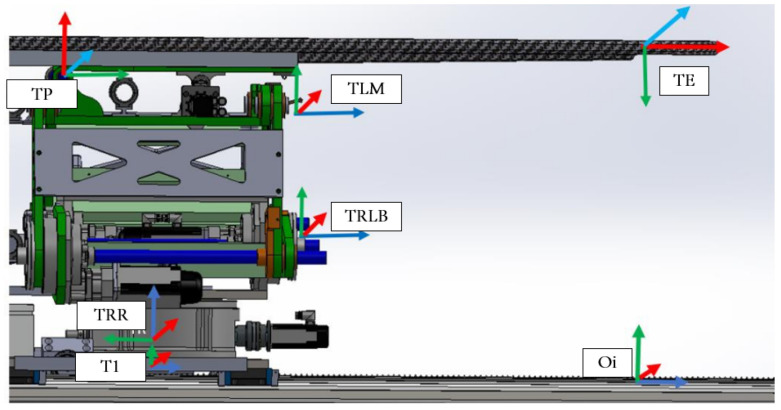
PPS frame assignment.

The patient positioning system (PPS) utilizes specific frame assignments to capture its distinct components and their movements, as shown in Figure 5:

- Base Frame ($O_{i}$): Located at the junction of the main rails.
- Linear Rail Frame ($T_{\mathrm{RR}}$): Positioned at the end of the linear rail.
- Lower Linkage System Frame ($T_{\mathrm{RLB}}$): At the midpoint of the lower linkage arm.
- Upper Linkage System Frame ($T_{\mathrm{LM}}$): At the midpoint of the upper linkage arm.
- Table Rod Frame ($T_{P}$): Centered on the table rod.
- Tabletop Frame ($T_{E}$): Centered on the tabletop.

**Figure 6:**
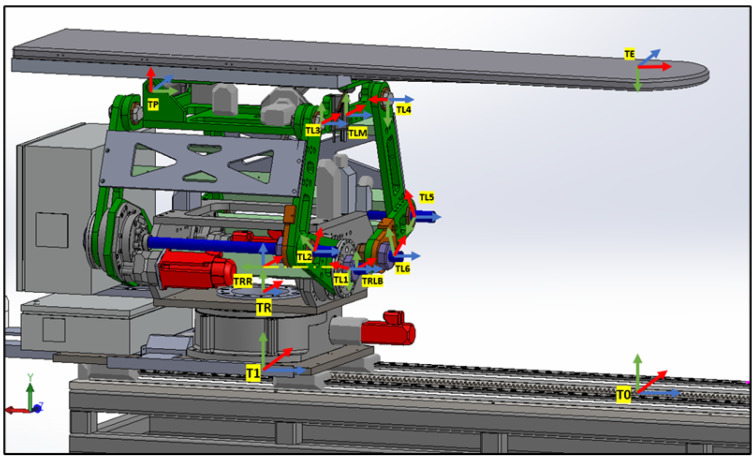
PPS frame assignment with (linkage frames).

The linkage frames are shown in Figure 6. These frame assignments are essential for precise kinematic evaluations in the PPS.

#### 4.1.1. Linear Rail System from T0 to TRR

**Figure 7:**
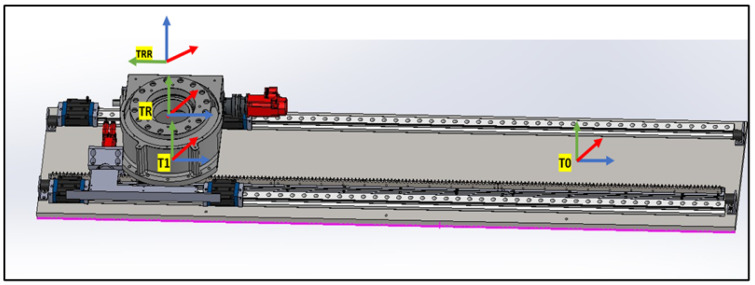
Linear Rail frame assignment (2).

This subsystem governs the bidirectional movement of the PPS. It facilitates both the translational motion of the main plate relative to the base plate along a rail system, and the rotational movement of the Linkage System around the main plate, courtesy of a rotary table as shown in Figure 7. The linear motion enables the patient to be safely introduced and withdrawn from the operational area. In rooms equipped with a CT scanner, the rotary table allows a complete 180° rotation of the patient for imaging purposes. Encoded motors drive both movements, ensuring the precise positioning required for such medical devices.

#### 4.1.2. Linkage System from TRLB to TLM

**Figure 8:**
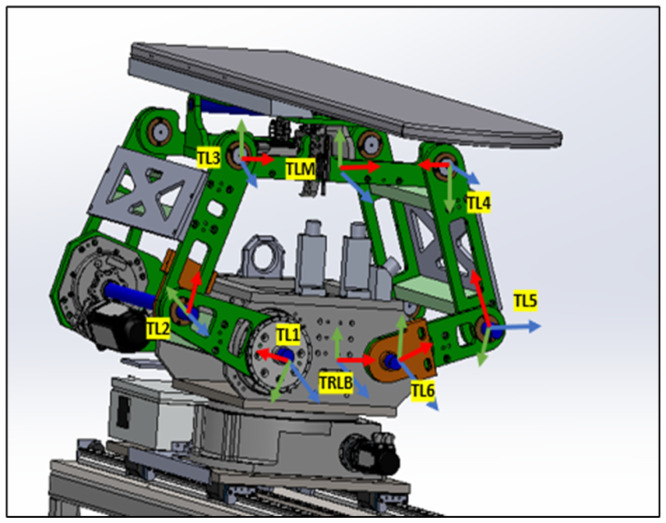
Linkage frame assignment.

Representing the heart of the PPS, the Linkage System comprises four pairs of two-arm linkages that connect the PPS to the plate on the rotary table. As shown in Figure 8, to maintain patient stability and alignment, precision shafts interconnect two of these four pairs. The system harnesses the power of three encoded motors to maneuver three joints, positioning the PPS within a 2D plane that spans a substantial envelope size. This robust design enables the table to be adjusted vertically to accommodate patient needs, from a lowered position facilitating access for elderly patients, to an elevated state suitable for treatment procedures [21].

#### 4.1.3. Table Tob TLM to TE

**Figure 9:**
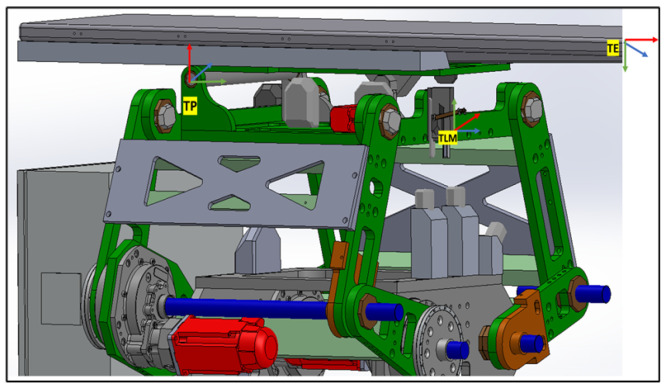
Table Top frame assignment.

Located at the top of the system, the Table Assembly is linked to the top plate of the Linkage Assembly. As shown in Figure 9, the table, a product of Siemens, features a carbon fiber exterior filled with resin to minimize radio interference during treatment. Given the cantilevered design of the tabletop and the associated gravitational deflection, a pitch adjustment mechanism has been integrated into the Table Assembly. This mechanism actively counteracts gravity, ensuring a consistently level operating area.

### 4.2. Formulating a Mathematical Model

#### 4.2.1. Linkage Subsystem Mathematical Modeling

**Figure 10:**
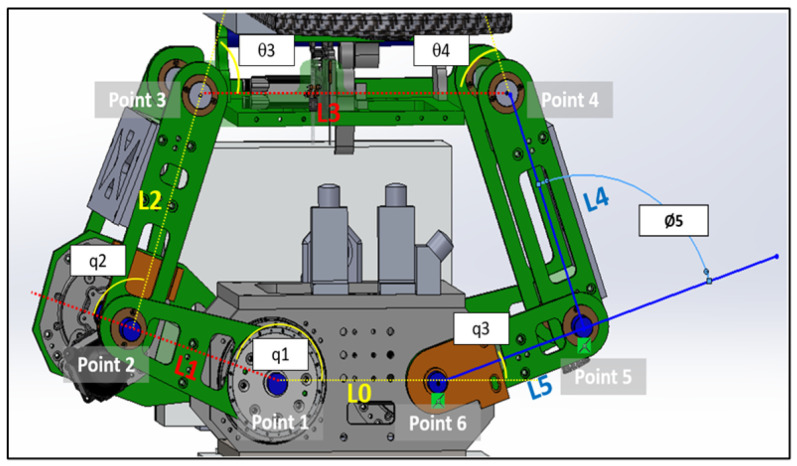
Linkage subsystems joints, lengths and angles.

This section primarily focuses on using geometric principles to develop a kinematic model for the linkage subsystem. Figure 10 illustrates the points, link lengths, and joint angles associated with this system [21].

We assumed that all the angles are measured counterclockwise:

- $q_{1}=0$ when L1 is on L0.
- $q_{2}=0$ when L2 is along the same line of L1.
- $q_{3}=0$ when L5 is along the same line with L0.
- $\theta_{3}=0$ when L3 is along the same line of L2.
- $\theta_{4}=0$ when L3 is along the same line of L4.
- $\theta_{5}=0$ when L4 is along the same line of L5.

We specify a coordinate frame for each joint as follows:

(4) $$\left(\begin{array}{c} x_{0}\\ y_{0} \end{array}\right)=\left(\begin{array}{cc} \cos q1 & -\sin q1\\ \sin q1 & \cos q1 \end{array}\right)\left(\begin{array}{c} x_{1}\\ y_{1} \end{array}\right)+\left(\begin{array}{c} 0\\ 0 \end{array}\right)$$

(5) $$\left(\begin{array}{c} x_{1}\\ y_{1} \end{array}\right)=\left(\begin{array}{cc} \cos q2 & -\sin q2\\ \sin q2 & \cos q2 \end{array}\right)\left(\begin{array}{c} x_{2}\\ y_{2} \end{array}\right)+\left(\begin{array}{c} L1\\ 0 \end{array}\right)$$

(6) $$\left(\begin{array}{c} x_{2}\\ y_{2} \end{array}\right)=\left(\begin{array}{cc} \cos\theta_{3} & -\sin\theta_{3}\\ \sin\theta_{3} & \cos\theta_{3} \end{array}\right)\left(\begin{array}{c} x_{3}\\ y_{3} \end{array}\right)+\left(\begin{array}{c} L2\\ 0 \end{array}\right)$$

where

(7) $$\left(\begin{array}{c} x_{0}\\ y_{0} \end{array}\right)=\left(\begin{array}{cc} \cos q3 & -\sin q3\\ \sin q3 & \cos q3 \end{array}\right)\left(\begin{array}{c} x_{6}\\ y_{6} \end{array}\right)+\left(\begin{array}{c} L0\\ 0 \end{array}\right)$$

(8) $$\left(\begin{array}{c} x_{6}\\ y_{6} \end{array}\right)=\left(\begin{array}{cc} \cos\theta_{5} & -\sin\theta_{5}\\ \sin\theta_{5} & \cos\theta_{5} \end{array}\right)\left(\begin{array}{c} x_{5}\\ y_{5} \end{array}\right)+\left(\begin{array}{c} L5\\ 0 \end{array}\right)$$

(9) $$\left(\begin{array}{c} x_{5}\\ y_{5} \end{array}\right)=\left(\begin{array}{cc} \cos\theta_{4} & -\sin\theta_{4}\\ \sin\theta_{4} & \cos\theta_{4} \end{array}\right)\left(\begin{array}{c} x_{4}\\ y_{4} \end{array}\right)+\left(\begin{array}{c} L4\\ 0 \end{array}\right)$$

(10) $$\left(\begin{array}{c} x_{4}\\ y_{4} \end{array}\right)=\left(\begin{array}{cc} \cos180 & -\sin180\\ \sin180 & \cos180 \end{array}\right)\left(\begin{array}{c} x_{3}\\ y_{3} \end{array}\right)+\left(\begin{array}{c} L3\\ 0 \end{array}\right)$$

From Equations (4) to (10) and Figure 10, we determined each point position as follows:

**Point 1**

(11) $${\left(\begin{array}{c} x_{0}\\ y_{0} \end{array}\right)}_{1}=\left(\begin{array}{cc} \cos q1 & -\sin q1\\ \sin q1 & \cos q1 \end{array}\right)\left(\begin{array}{c} 0\\ 0 \end{array}\right)+\left(\begin{array}{c} 0\\ 0 \end{array}\right)=\left(\begin{array}{c} 0\\ 0 \end{array}\right)$$

**Point 2**

(12) $${\left(\begin{array}{c} x_{0}\\ y_{0} \end{array}\right)}_{2}=\left(\begin{array}{cc} \cos q1 & -\sin q1\\ \sin q1 & \cos q1 \end{array}\right)\left(\begin{array}{c} L1\\ 0 \end{array}\right)=\left(\begin{array}{c} L1\cos q1\\ L1\sin q1 \end{array}\right)$$

**Point 3**

(13) $${\left(\begin{array}{c} x_{1}\\ y_{1} \end{array}\right)}_{3}=\left(\begin{array}{cc} \cos q2 & -\sin q2\\ \sin q2 & \cos q2 \end{array}\right)\left(\begin{array}{c} L2\\ y_{2} \end{array}\right)+\left(\begin{array}{c} L1\\ 0 \end{array}\right)=\left(\begin{array}{c} L2\cos q2+L1\\ L2\sin q2 \end{array}\right)$$

(14) $$\begin{array}{cc} {\left(\begin{array}{c} x_{0}\\ y_{0} \end{array}\right)}_{3}\; & =\left(\begin{array}{cc} \cos q1 & -\sin q1\\ \sin q1 & \cos q1 \end{array}\right)\left(\begin{array}{c} L2\cos q2+L1\\ L2\sin q2 \end{array}\right)\\ & =\left(\begin{array}{c} L2\cos q1\cos q2+L1\cos q1-L2\sin q1\sin q2\\ L2\sin q1\cos q2+L1\sin q1+L2\cos q1\sin q2 \end{array}\right) \end{array}$$

**Point 6**

(15) $${\left(\begin{array}{c} x_{0}\\ y_{0} \end{array}\right)}_{6}=\left(\begin{array}{cc} \cos q3 & -\sin q3\\ \sin q3 & \cos q3 \end{array}\right)\left(\begin{array}{c} 0\\ 0 \end{array}\right)+\left(\begin{array}{c} L0\\ 0 \end{array}\right)=\left(\begin{array}{c} L0\\ 0 \end{array}\right)$$

**Point 5**

(16) $${\left(\begin{array}{c} x_{6}\\ y_{6} \end{array}\right)}_{5}=\left(\begin{array}{cc} \cos\theta_{5} & -\sin\theta_{5}\\ \sin\theta_{5} & \cos\theta_{5} \end{array}\right)\left(\begin{array}{c} 0\\ 0 \end{array}\right)+\left(\begin{array}{c} L5\\ 0 \end{array}\right)=\left(\begin{array}{c} L5\\ 0 \end{array}\right)$$

(17) $${\left(\begin{array}{c} x_{0}\\ y_{0} \end{array}\right)}_{5}=\left(\begin{array}{cc} \cos q3 & -\sin q3\\ \sin q3 & \cos q3 \end{array}\right)\left(\begin{array}{c} L5\\ 0 \end{array}\right)+\left(\begin{array}{c} L0\\ 0 \end{array}\right)=\left(\begin{array}{c} L5\cos q3+L0\\ L5\sin q3 \end{array}\right)$$

Therefore, **Point 4**

(18) $${\left(\begin{array}{c} x_{5}\\ y_{5} \end{array}\right)}_{4}=\left(\begin{array}{cc} \cos\theta_{4} & -\sin\theta_{4}\\ \sin\theta_{4} & \cos\theta_{4} \end{array}\right)\left(\begin{array}{c} 0\\ 0 \end{array}\right)+\left(\begin{array}{c} L4\\ 0 \end{array}\right)=\left(\begin{array}{c} L4\\ 0 \end{array}\right)$$

(19) $${\left(\begin{array}{c} x_{6}\\ y_{6} \end{array}\right)}_{4}=\left(\begin{array}{cc} \cos\theta_{5} & -\sin\theta_{5}\\ \sin\theta_{5} & \cos\theta_{5} \end{array}\right)\left(\begin{array}{c} L4\\ 0 \end{array}\right)+\left(\begin{array}{c} L5\\ 0 \end{array}\right)\;=\;\left(\begin{array}{c} L4\cos\theta_{5}+L5\\ L4\sin\theta_{5} \end{array}\right)$$

(20) x0y04=cosq3−sinq3sinq3cosq3L4cosθ5+L5L4sinθ5+L00=L4cosq3cosθ5+L5cosq3−L4sinq3sinθ5+L0L4sinq3cosθ5+L5sinq3+L4cosq3sinθ5

#### 4.2.2. Intersection of Two Circles

**Figure 11:**
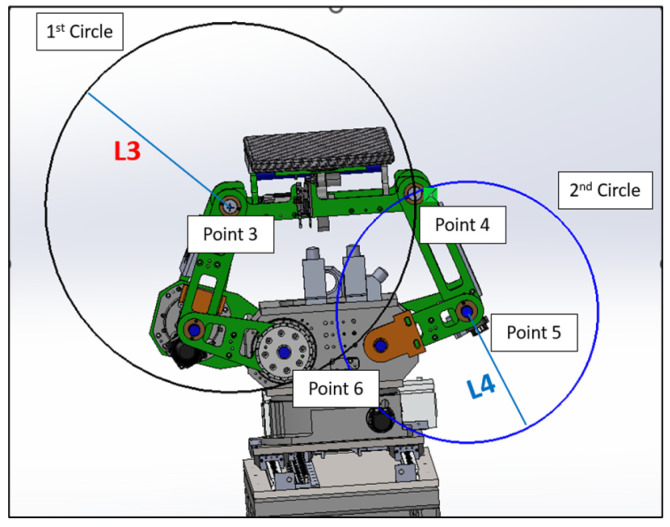
Two circles intersection.

In the domain of kinematics and mechanical geometry, a well-accepted technique for determining the location of a specific point within a linkage mechanism involves the intersection of two circles [25]. As shown in Figure 11, we can pinpoint the position of Point 4 using this intersecting circles methodology. The scenario involves two circles: the first centered at Point 3 with a radius of L3, and the second centered at Point 5 with a radius of L5. The intersection of these two circles yields two potential points, namely Point 4 and Point 6.

Consider two circles:

- Circle 1, centered at Point 3 ($C_{3}=\left(x_{3},y_{3}\right)$) with radius $L_{3}$.
- Circle 2, centered at Point 5 ($C_{5}=\left(x_{5},y_{5}\right)$) with radius $L_{5}$.

The equations of these circles are as follows:

(21) $${\left(x-x_{3}\right)}^{2}+{\left(y-y_{3}\right)}^{2}=L_{3}^{2}$$

(22) $${\left(x-x_{5}\right)}^{2}+{\left(y-y_{5}\right)}^{2}=L_{5}^{2}$$

To find the intersection points, we can subtract two circle equations and simplify these. This will give a linear equation in *x* and *y*.

Out of the two intersection points, the one with the greater *y*-coordinate is considered the upper intersection point. We choose this as Point 4, and the other intersection point is disregarded.

These represent the possible locations of the mechanical joint or linkage. However, due to the physical constraints and design of our mechanical system, the existence of Point 6 would suggest a mechanically unfeasible configuration. Therefore, Point 6 can be categorically eliminated from our options, leaving us with the valid position, Point 4.

**Figure 12:**
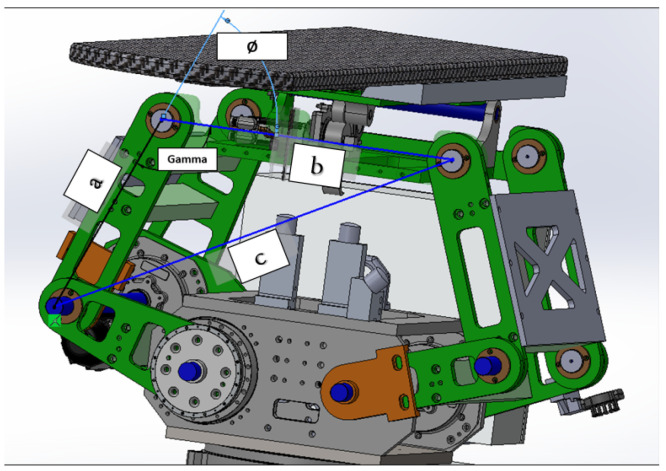
Upper joint angle calculation.

After that, to ascertain the orientation of the upper arm of the linkage mechanism Figure 12, we must calculate the value of $\phi_{3}$. This angle $\phi_{3}$ is a critical parameter that provides insights into the inclination of the upper arm, thereby enabling a comprehensive understanding of the system’s kinematics. The determination of $\phi_{3}$ is a crucial step in accurately modeling and analyzing the mechanical system.

(23) $$c=\sqrt{{\left(P4\left(x\right)-P2\left(x\right)\right)}^{2}+{\left(P4\left(y\right)-P2\left(y\right)\right)}^{2}}$$

(24) $$\Gamma =\mathrm{arc}\left(\frac{a^{2}+b^{2}-c^{2}}{2\times a\times b}\right)$$

(25) $$\phi_{3}=180^{\circ }-\Gamma$$

### 4.3. Table Pitching Mathematical Modeling

**Figure 13:**
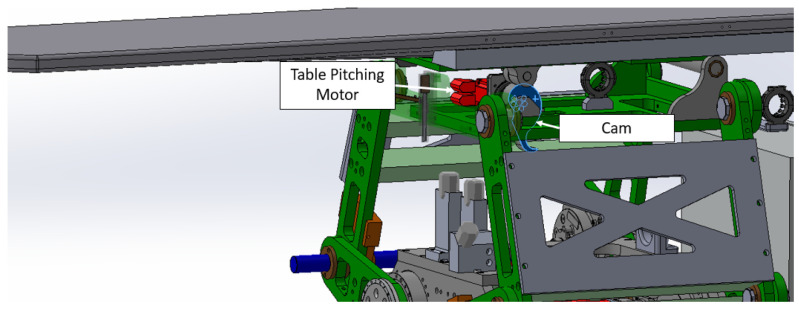
Table pitching design.

Table angle or pitching angle controlled by servo motor and CAM as shown in the following Figure 13.

**Figure 14:**
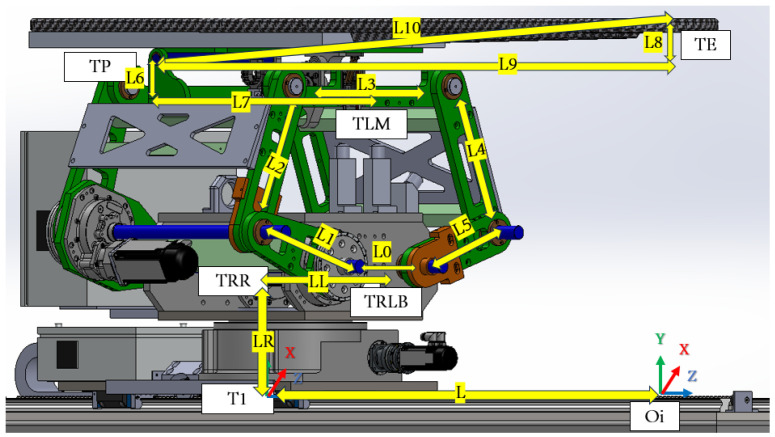
PPS length parameters needed for DH.

In order to transition from point TP to point TE, it is necessary to calculate the length L10, based on the angle input as per the given Table 1 and Figure 14.

Then, after determining all needed parameters from Figure 14, we can create the DH table as follows:

**Table 1:** DH parameters for the linkage from the origin frame to the endpoint frame.

Frame	$a_{i}$	α	$d_{i}$	θ
Linear Rail subsystem				
T0	0	0	0	0
T1	0	0	−L+Lin	0
TR	0	−π/2	0	0
TRR	0	0	LR	Rot
Linkage subsystem				
TRLB	0	π/2	0	0
TL1	L0/2	0	LL	π
TL2	L1	0	0	−π+q1
TL3	L2	0	0	q2
TLM	L3/2	0	0	$\theta_{3}$
Table Top subsystem				
TP	L6	π/2	L7	π/2
TE	L10	0	0	$\pi /2+\mathrm{Table}\;\mathrm{Angle}-\theta_{6}$
Euler rotation matrices				
TE1	0	0	0	$-\pi /2-\theta_{6}$
TE2	0	−π/2	0	0
TE3	0	0	0	−π/2

### 4.4. Utilize the Denavit–Hartenberg (DH) Parameters

With the essential parameters and points now determined, we can utilize the Denavit–Hartenberg (DH) parameters. This allows us to transition from one frame to the subsequent frame, ultimately leading us to the final transformation matrix representing our PPS.

#### 4.4.1. DH Matrix Formulation

The standard DH transformation matrix, denoted as $A_{i}$, is constructed using the given parameters *a*, α, *d*, and θ [23]:

$$A_{i}=\left[\begin{array}{cccc} \cos(\theta ) & -\sin(\theta ) & 0 & 0\\ \sin(\theta ) & \cos(\theta ) & 0 & 0\\ 0 & 0 & 1 & 0\\ 0 & 0 & 0 & 1 \end{array}\right]\left[\begin{array}{cccc} 1 & 0 & 0 & 0\\ 0 & 1 & 0 & 0\\ 0 & 0 & 1 & d\\ 0 & 0 & 0 & 1 \end{array}\right]\left[\begin{array}{cccc} 1 & 0 & 0 & a\\ 0 & 1 & 0 & 0\\ 0 & 0 & 1 & 0\\ 0 & 0 & 0 & 1 \end{array}\right]\left[\begin{array}{cccc} 1 & 0 & 0 & 0\\ 0 & \cos(\alpha ) & -\sin(\alpha ) & 0\\ 0 & \sin(\alpha ) & \cos(\alpha ) & 0\\ 0 & 0 & 0 & 1 \end{array}\right]$$

#### 4.4.2. Linear Rail Subsystem

The transformation matrix for the linear rail subsystem, denoted as $T_{o}$, is as follows:

$$T_{o}=A_{i}|{\,}_{\begin{array}{c} a=0,\\ \alpha =0,\\ d=0,\\ \theta =0 \end{array}}$$

$$T_{1}=A_{i}|{\,}_{\begin{array}{c} a=0,\\ \alpha =0,\\ d=-L+Lin,\\ \theta =0 \end{array}}$$

(26) $$T_{0}^{1}=\left[\begin{array}{cccc} 1 & 0 & 0 & 0\\ 0 & 1 & 0 & 0\\ 0 & 0 & 1 & \mathrm{Lin}-L\\ 0 & 0 & 0 & 1 \end{array}\right]$$

**Figure 15:**
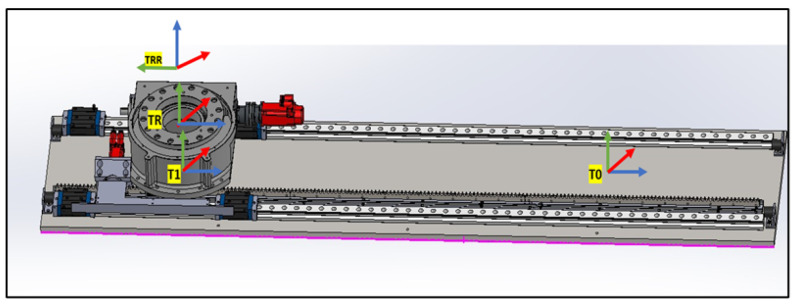
Linear Rail frames.

As shown in Figure 15, the transformation matrices describe the motion and positioning of the linear rail system.

#### 4.4.3. Rotary Base to the Height of the Linkage

**Figure 16:**
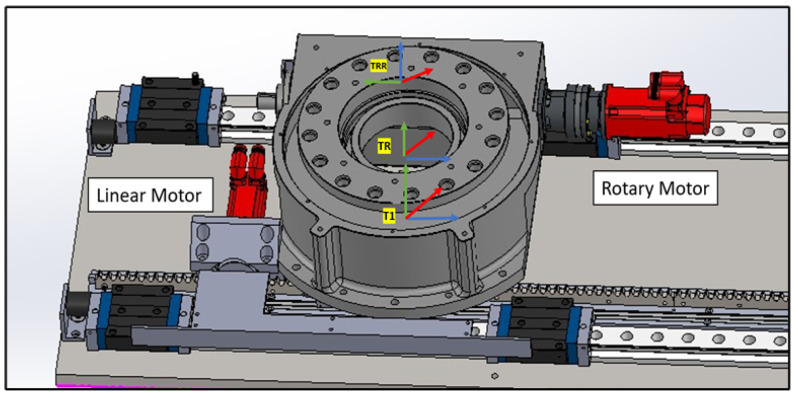
Linear Rail frame assignment.

As shown in Figure 16, two transformation matrices are derived:

1. $T_{R}$ representing a rotation about the θ-axis by $-\frac{\pi }{2}$ and no translation along the *x*-axis:
  $$T_{R}=A_{i}|{\,}_{\begin{array}{c} a=0,\\ \alpha =0,\\ d=0,\\ \theta =-\frac{\pi }{2} \end{array}}$$
2. $T_{RR}$ representing a rotation about the ‘Rott’ angle and a translation along the *z*-axis by LR:
  $$T_{RR}=A_{i}|{\,}_{\begin{array}{c} a=0,\\ \alpha =0,\\ d=LR,\\ \theta =Rot \end{array}}$$
  (27) $$T_{R}^{RR}=\left[\begin{array}{cccc} 1 & 0 & 0 & 0\\ 0 & 0 & 1 & 0\\ 0 & -1 & 0 & 0\\ 0 & 0 & 0 & 1 \end{array}\right]$$
  (28) $$T_{RR}^{RLB}=\left[\begin{array}{cccc} \cos(Rot) & -\sin(Rot) & 0 & 0\\ \sin(Rot) & \cos(Rot) & 0 & 0\\ 0 & 0 & 1 & LR\\ 0 & 0 & 0 & 1 \end{array}\right]$$

#### 4.4.4. Linkage Subsystem

The linkage subsystem of the PPS consists of multiple stages of transformation:

1. First, no translation along the *x*-axis and a rotation of $\frac{\pi }{2}$ about the *x*-axis:
  $$T_{RLB}=A_{i}|{\,}_{\begin{array}{c} a=0,\\ \alpha =\frac{\pi }{2},\\ d=0,\\ \theta =0 \end{array}}$$
  (29) $$T_{RLB}^{L1}=\left[\begin{array}{cccc} 1 & 0 & 0 & 0\\ 0 & 0 & -1 & 0\\ 0 & 1 & 0 & 0\\ 0 & 0 & 0 & 1 \end{array}\right]$$
2. Second, a translation of $\frac{L0}{2}$ along the *x*-axis, a translation of LL along the *z*-axis, and a rotation of π about the *z*-axis:
  $$T_{L1}=A_{i}|{\,}_{\begin{array}{c} a=\frac{L0}{2},\\ \alpha =0,\\ d=LL,\\ \theta =\{\pi \end{array}}$$
3. Next, we move the first link by controlling the first motor q1. This involves a translation of L1 along the *x*-axis and a rotation of −π+q1 about the *z*-axis:
  $$T_{L2}=A_{i}|{\,}_{\begin{array}{c} a=L1,\\ \alpha =0,\\ d=0,\\ \theta =pi+q1 \end{array}}$$
  (30) $$T_{L1}^{L2}=\left[\begin{array}{cccc} -1 & 0 & 0 & -\frac{L0}{2}\\ 0 & -1 & 0 & 0\\ 0 & 0 & 1 & LL\\ 0 & 0 & 0 & 1 \end{array}\right]$$
4. After this, we control the second link with the second motor q2. This step comprises a translation of L2 along the *x*-axis and a rotation of q2 about the *z*-axis:
  $$T_{L12}=A_{i}|{\,}_{\begin{array}{c} a=L2,\\ \alpha =0,\\ d=0,\\ \theta =q2 \end{array}}$$
  (31) $$T_{L3}^{LM}=\left[\begin{array}{cccc} \cos(q2) & -\sin(q2) & 0 & L2\cos(q2)\\ \sin(q2) & \cos(q2) & 0 & L2\sin(q2)\\ 0 & 0 & 1 & 0\\ 0 & 0 & 0 & 1 \end{array}\right]$$
5. Lastly, we move to the middle of the upper arm based on the ϕ3 value. This involves a translation of $\frac{L3}{2}$ along the *x*-axis and a rotation of ϕ3 about the *z*-axis:
  $$T_{LM}=A_{i}|{\,}_{\begin{array}{c} a=\frac{L3}{2},\\ \alpha =0,\\ d=0,\\ \theta =\phi 3 \end{array}}$$
  (32) $$T_{LM}^{P}=\left[\begin{array}{cccc} \cos(\theta_{3}) & -\sin(\theta_{3}) & 0 & \frac{L3\cos(\theta_{3})}{2}\\ \sin(\theta_{3}) & \cos(\theta_{3}) & 0 & \frac{L3\sin(\theta_{3})}{2}\\ 0 & 0 & 1 & 0\\ 0 & 0 & 0 & 1 \end{array}\right]$$

#### 4.4.5. Moving to the Table Pitching Frame

See Figure 9 for an illustration of the following transition. We transition from $T_{LM}$ to $T_{P}$. This involves a translation of L6 along the *x*-axis, a rotation of $\frac{\pi }{2}$ about the *z*-axis, a translation of L7 along the *z*-axis, and another rotation of $\frac{\pi }{2}$ about the *z*-axis:

$$T_{P}=A_{i}|{\,}_{\begin{array}{c} a=L6,\\ \alpha =\frac{\pi }{2},\\ d=L7,\\ \theta =\frac{\pi }{2} \end{array}}$$

(33) $$T_{P}^{E}=\left[\begin{array}{cccc} 0 & 0 & 1 & 0\\ 1 & 0 & 0 & L6\\ 0 & 1 & 0 & L7\\ 0 & 0 & 0 & 1 \end{array}\right]$$

#### 4.4.6. Transitioning from the Pitching Axis to the Endpoint

**Figure 17:**
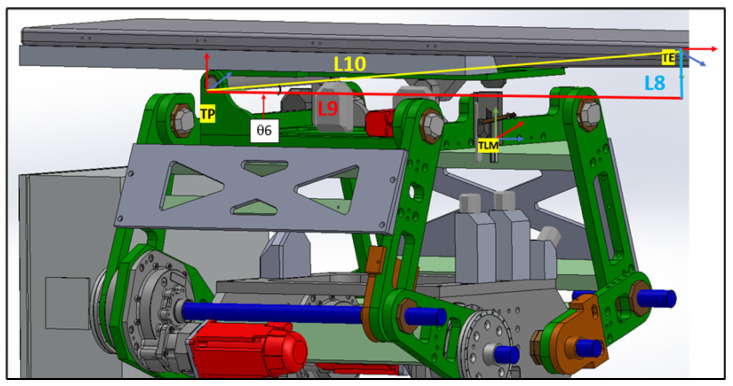
Moving from table pitching axis to endpoint.

As shown in Figure 17, we use $T_{PE}$. The translation along the *x*-axis is determined by the hypotenuse of L9 and L8. The rotation about the *z*-axis is defined as $\frac{\pi }{2}$ minus the table angle (‘Tabang’) and the arctangent of the ratio $\frac{L9}{L8}$. This angle is derived from the pitching encoder:

$$T_{PE}=A_{i}|{\,}_{\begin{array}{c} a=\sqrt{{\left(L9\right)}^{2}+{\left(L8\right)}^{2}},\\ \alpha =0,\\ d=0,\\ \theta =\left(\frac{\pi }{2}-\mathrm{Tabang}-\arctan\left(\frac{L9}{L8}\right)\right) \end{array}}$$

(34) $$T_{E}^{E1}=\left[\begin{array}{cccc} \cos(\mathrm{TabAng}-\theta_{6}+\frac{\pi }{2}) & -\sin(\mathrm{TabAng}-\theta_{6}+\frac{\pi }{2}) & 0 & L10\cos(\mathrm{TabAng}-\theta_{6}+\frac{\pi }{2})\\ \sin(\mathrm{TabAng}-\theta_{6}+\frac{\pi }{2}) & \cos(\mathrm{TabAng}-\theta_{6}+\frac{\pi }{2}) & 0 & L10\sin(\mathrm{TabAng}-\theta_{6}+\frac{\pi }{2})\\ 0 & 0 & 1 & 0\\ 0 & 0 & 0 & 1 \end{array}\right]$$

#### 4.4.7. Additional Frames Are Integrated to Align with the Reference Direction

In Figure 17, it is evident that the TE coordinate does not align with the reference coordinate shown in Figure 5. To correct this orientation, several rotational matrices need to be applied as follows:

- TE1: Rotation about the *z*-axis is determined by $-\frac{\pi }{2}$ minus the arctangent of the ratio $\frac{L9}{L8}$. No translations are considered for this frame.
  $$TE1=A_{i}|{\,}_{\begin{array}{c} a=0,\\ \alpha =0,\\ d=0,\\ \theta =-\left(-\frac{\pi }{2}\arctan\left(\frac{L9}{L8}\right)\right) \end{array}}$$
- TE2: A rotation of $-\frac{\pi }{2}$ about the *x*-axis. No translations or other rotations are involved.
  $$TE2=A_{i}|{\,}_{\begin{array}{c} a=0,\\ \alpha =-\frac{\pi }{2},\\ d=0,\\ \theta =0 \end{array}}$$
- TE: A rotation of $-\frac{\pi }{2}$ about the *z*-axis. Again, no translations or other rotations are considered.
  $$TE=A_{i}|{\,}_{\begin{array}{c} a=0,\\ \alpha =0,\\ d=0,\\ \theta =-\frac{\pi }{2} \end{array}}$$

(35) $$T_{E1}^{E2}=\left[\begin{array}{cccc} \cos(\theta_{6}-\frac{\pi }{2}) & -\sin(\theta_{6}-\frac{\pi }{2}) & 0 & 0\\ \sin(\theta_{6}-\frac{\pi }{2}) & \cos(\theta_{6}-\frac{\pi }{2}) & 0 & 0\\ 0 & 0 & 1 & 0\\ 0 & 0 & 0 & 1 \end{array}\right]$$

(36) $$T_{E2}^{E3}=\left[\begin{array}{cccc} 1 & 0 & 0 & 0\\ 0 & 0 & 1 & 0\\ 0 & -1 & 0 & 0\\ 0 & 0 & 0 & 1 \end{array}\right]$$

(37) $$T_{E3}^{0}=\left[\begin{array}{cccc} 0 & 1 & 0 & 0\\ -1 & 0 & 0 & 0\\ 0 & 0 & 1 & 0\\ 0 & 0 & 0 & 1 \end{array}\right]$$

Table 1 outlines the Denavit–Hartenberg (DH) parameters for the PPS system. These parameters are essential for representing the kinematic transformations between adjacent frames of the system. Each row describes a specific transformation with the respective $a_{i}$, $\alpha_{i}$, $d_{i}$, and $\theta_{i}$ parameters, as stipulated by Equation (3).

(38) $$T_{o}^{E}=T_{o}\cdot T_{1}\cdot T_{R}\cdot T_{RR}\cdot T_{RLB}\cdot T_{L1}\cdot T_{L2}\cdot T_{L3}\cdot T_{LM}\cdot T_{P}\cdot T_{E}\cdot T_{E1}\cdot T_{E2}\cdot T_{E3}$$

The 4×4 matrix $T_{o}^{E}$ is a homogeneous transformation matrix commonly used in the field of robotics and computer graphics to represent both the position and orientation of a body in space.

The matrix $T_{o}^{E}$ is given by [26]:

$$T_{o}^{E}=\left[\begin{array}{cccc} \mu_{x} & O_{x} & \alpha_{x} & p_{x}\\ \mu_{y} & O_{y} & \alpha_{y} & p_{y}\\ \mu_{z} & O_{z} & \alpha_{z} & p_{z}\\ 0 & 0 & 0 & 1 \end{array}\right]$$

1. **Position (Translation Vector)**:
  The elements $p_{x}$, $p_{y}$, and $p_{z}$ in the fourth column represent the position of the target point with respect to the origin. They give the *x*, *y*, and *z* coordinates of the point in the base or reference frame. In the context of robotics, this would be the position of the end effector or tool tip relative to the base or reference frame.
2. **Orientation (Rotation Matrix)**:
  - The 3×3 matrix on the top-left corner of $T_{o}^{E}$ represents the orientation of the body in space. The columns
    $$\left[\begin{array}{c} \mu_{x}\\ \mu_{y}\\ \mu_{z} \end{array}\right],\left[\begin{array}{c} O_{x}\\ O_{y}\\ O_{z} \end{array}\right],$$
    and
    $$\left[\begin{array}{c} \alpha_{x}\\ \alpha_{y}\\ \alpha_{z} \end{array}\right]$$
    are unit vectors that represent the orientation of the body’s local x, y, and z axes, respectively, in the base frame [27].
  - These vectors are often called the rotation axes, and their magnitude is always 1. The orientation of the body can be described using various representations like Euler angles, rotation matrices, or quaternions. In this matrix format, the orientation is represented using the rotation matrix [28]

We can extract the Euler angles from the following

(39) $$R_{\mathrm{xzy}}=R_{y}\cdot R_{z}\cdot R_{x}$$

(40) $$R_{\mathrm{xzy}}=\left[\begin{array}{ccc} \cos\beta \cos\gamma & -\cos\beta \sin\gamma \cos\alpha +\sin\beta \sin\alpha & \cos\beta \sin\gamma \sin\alpha +\sin\beta \cos\alpha \\ \sin\gamma & \cos\gamma \cos\alpha & -\cos\gamma \sin\alpha \\ -\sin\beta \cos\gamma & \sin\beta \sin\gamma \cos\alpha +\cos\beta \sin\alpha & -\sin\beta \sin\gamma \sin\alpha +\cos\beta \cos\alpha \end{array}\right]$$

Through a substitution in the matrices of each link and by multiplying these, we obtain the final matrix which gives the information for both motions as follows:

(41) $$\mu_{x}=\cos\left(q1+q2+\theta_{3}\right)\times \cos\left(Rot\right)$$

(42) $$\mu_{y}=\sin\left(q1+q2+\theta_{3}\right)$$

(43) $$\mu_{z}=-\cos\left(q1+q2+\theta_{3}\right)\times \sin\left(\mathrm{Rot}\right)$$

(44) $$\begin{array}{cc} O_{x} & =\sin(\mathrm{Rot})\times \sin(\mathrm{TabAng})\\ \; & \;-\cos\left(\mathrm{Rot}\right)\times \cos\left(\mathrm{TabAng}\right)\times \cos\left(q1\right)\times \cos\left(q2\right)\times \sin\left(\theta_{3}\right)\\ \; & \;-\cos\left(\mathrm{Rot}\right)\times \cos\left(\mathrm{TabAng}\right)\times \cos\left(q1\right)\times \cos\left(\theta_{3}\right)\times \sin\left(q2\right)\\ \; & \;-\cos\left(\mathrm{Rot}\right)\times \cos\left(\mathrm{TabAng}\right)\times \cos\left(q2\right)\times \cos\left(\theta_{3}\right)\times \sin\left(q1\right)\\ \; & \;+\cos\left(\mathrm{Rot}\right)\times \cos\left(\mathrm{TabAng}\right)\times \sin\left(q1\right)\times \sin\left(q2\right)\times \sin\left(\theta_{3}\right) \end{array}$$

(45) $$O_{y}=\frac{\cos(\mathrm{TabAng}+q1+q2+\theta_{3})}{2}+\frac{\cos(q1-\mathrm{TabAng}+q2+\theta_{3})}{2}$$

(46) $$\begin{array}{cc} O_{z} & =\cos(\mathrm{Rot})\cdot \sin(\mathrm{TabAng})\\ \; & \;+\cos\left(\mathrm{TabAng}\right)\cdot \sin\left(\mathrm{Rot}\right)\cdot \cos\left(q1\right)\cdot \cos\left(q2\right)\cdot \sin\left(\theta_{3}\right)\\ \; & \;+\cos\left(\mathrm{TabAng}\right)\cdot \sin\left(\mathrm{Rot}\right)\cdot \cos\left(q1\right)\cdot \cos\left(\theta_{3}\right)\cdot \sin\left(q2\right)\\ \; & \;+\cos\left(\mathrm{TabAng}\right)\cdot \sin\left(\mathrm{Rot}\right)\cdot \cos\left(q2\right)\cdot \cos\left(\theta_{3}\right)\cdot \sin\left(q1\right)\\ \; & \;-\cos\left(\mathrm{TabAng}\right)\cdot \sin\left(\mathrm{Rot}\right)\cdot \sin\left(q1\right)\cdot \sin\left(q2\right)\cdot \sin\left(\theta_{3}\right) \end{array}$$

(47) $$\begin{array}{cc} a_{x} & =\cos(\mathrm{TabAng})\cdot \sin(\mathrm{Rot})\\ \; & \;+\cos\left(\mathrm{Rot}\right)\cdot \sin\left(\mathrm{TabAng}\right)\cdot \cos\left(q1\right)\cdot \cos\left(q2\right)\cdot \sin\left(\theta_{3}\right)\\ \; & \;+\cos\left(\mathrm{Rot}\right)\cdot \sin\left(\mathrm{TabAng}\right)\cdot \cos\left(q1\right)\cdot \cos\left(\theta_{3}\right)\cdot \sin\left(q2\right)\\ \; & \;+\cos\left(\mathrm{Rot}\right)\cdot \sin\left(\mathrm{TabAng}\right)\cdot \cos\left(q2\right)\cdot \cos\left(\theta_{3}\right)\cdot \sin\left(q1\right)\\ \; & \;-\cos\left(\mathrm{Rot}\right)\cdot \sin\left(\mathrm{TabAng}\right)\cdot \sin\left(q1\right)\cdot \sin\left(q2\right)\cdot \sin\left(\theta_{3}\right) \end{array}$$

(48) $$a_{y}=\frac{\sin(q1-\mathrm{TabAng}+q2+\theta_{3})}{2}-\frac{\sin(\mathrm{TabAng}+q1+q2+\theta_{3})}{2}$$

(49) $$\begin{array}{cc} a_{z} & =\cos(\mathrm{Rot})\cos(\mathrm{TabAng})\\ \; & \;-\sin\left(\mathrm{Rot}\right)\sin\left(\mathrm{TabAng}\right)\cos\left(q1\right)\cos\left(q2\right)\sin\left(\theta_{3}\right)\\ \; & \;-\sin\left(\mathrm{Rot}\right)\sin\left(\mathrm{TabAng}\right)\cos\left(q1\right)\cos\left(\theta_{3}\right)\sin\left(q2\right)\\ \; & \;-\sin\left(\mathrm{Rot}\right)\sin\left(\mathrm{TabAng}\right)\cos\left(q2\right)\cos\left(\theta_{3}\right)\sin\left(q1\right)\\ \; & \;+\sin\left(\mathrm{Rot}\right)\sin\left(\mathrm{TabAng}\right)\sin\left(q1\right)\sin\left(q2\right)\sin\left(\theta_{3}\right) \end{array}$$

(50) $$\begin{array}{cc} p_{x} & =L7\sin\left(\mathrm{Rot}\right)-\frac{L0\cos(\mathrm{Rot})}{2}+LL\sin\left(\mathrm{Rot}\right)\\ \; & \;+L1\cos\left(\mathrm{Rot}\right)\cos\left(q1\right)+L10\sin\left(\mathrm{Rot}\right)\cos\left(\mathrm{TabAng}-\theta_{6}\right)\\ \; & \;-L6\sin\left(q1+q2+\theta_{3}\right)\cos\left(\mathrm{Rot}\right)\\ \; & \;+L2\cos(\mathrm{Rot})\cos(q1)\cos(q2)-L2\cos(\mathrm{Rot})\sin(q1)\sin(q2)\\ \; & \;+L10\sin\left(q1+q2+\theta_{3}\right)\cos\left(\mathrm{Rot}\right)\sin\left(\mathrm{TabAng}-\theta_{6}\right)\\ \; & \;+\frac{L3\cos\left(q1+q2\right)\cos\left(\mathrm{Rot}\right)\cos\left(\theta_{3}\right)}{2}\\ \; & \;-\frac{L3\sin\left(q1+q2\right)\cos\left(\mathrm{Rot}\right)\sin\left(\theta_{3}\right)}{2} \end{array}$$

(51) $$\begin{array}{cc} p_{y} & =LR+L2\sin(q1+q2)+L1\sin(q1)\\ \; & \;+L6\cos(q1+q2+\theta_{3})\\ \; & \;+\frac{L3\sin(q1+q2+\theta_{3})}{2}\\ \; & \;-\frac{L10\sin(\mathrm{TabAng}+q1+q2+\theta_{3}-\theta_{6})}{2}\\ \; & \;+\frac{L10\sin(q1-\mathrm{TabAng}+q2+\theta_{3}+\theta_{6})}{2} \end{array}$$

(52) $$\begin{array}{cc} p_{z}= & \mathrm{lin}-L+L7\cos\left(\mathrm{Rot}\right)+LL\cos\left(\mathrm{Rot}\right)+\frac{L0\sin(\mathrm{Rot})}{2}\\ \; & -L1\sin\left(\mathrm{Rot}\right)\cos\left(q1\right)+L10\cos\left(\mathrm{Rot}\right)\cos\left(\mathrm{TabAng}-\theta_{6}\right)\\ \; & +L6\sin\left(q1+q2+\theta_{3}\right)\sin\left(\mathrm{Rot}\right)+\frac{L3\sin\left(q1+q2\right)\sin\left(\mathrm{Rot}\right)\sin\left(\theta_{3}\right)}{2}\\ \; & -L2\sin(\mathrm{Rot})\cos(q1)\cos(q2)+L2\sin(\mathrm{Rot})\sin(q1)\sin(q2)\\ \; & -L10\sin\left(q1+q2+\theta_{3}\right)\sin\left(\mathrm{Rot}\right)\sin\left(\mathrm{TabAng}-\theta_{6}\right)\\ \; & -\frac{L3\cos\left(q1+q2\right)\sin\left(\mathrm{Rot}\right)\cos\left(\theta_{3}\right)}{2} \end{array}$$

From Equation (39), we can extract Euler angles using (53)–(55).

(53) $$\begin{array}{cc} \alpha & =\tan^{-1}\left(\frac{\sin\alpha }{\cos\alpha }\right)\\ \; & =\tan^{-1}\left(\frac{\cos\gamma \cdot \sin\alpha }{\cos\alpha \cdot \cos\gamma }\right)\\ \; & =\tan^{-1}\left(\frac{-R_{\mathrm{xzy},2,3}}{R_{\mathrm{xzy},2,2}}\right)\\ \; & =\tan^{-1}\left(\frac{-R_{2,3}}{R_{2,2}}\right) \end{array}$$

(54) $$\begin{array}{cc} \beta & =\tan^{-1}\left(\frac{\sin\beta }{\cos\beta }\right)\\ \; & =\tan^{-1}\left(\frac{\cos\gamma \cdot \sin\beta }{\cos\beta \cdot \cos\gamma }\right)\\ \; & =\tan^{-1}\left(\frac{-R_{\mathrm{xzy},3,1}}{R_{\mathrm{xzy},1,1}}\right)\\ \; & =\tan^{-1}\left(\frac{R_{3,1}}{R_{1,1}}\right) \end{array}$$

(55) $$\begin{array}{cc} \gamma & =\tan^{-1}\left(\frac{\sin\gamma }{\cos\gamma }\right)\\ \; & =\tan^{-1}\left(\frac{\sin\gamma }{\sqrt{{\left(\cos\alpha \cdot \cos\gamma \right)}^{2}+{\left(-\cos\gamma \cdot \sin\alpha \right)}^{2}}}\right)\\ \; & =\tan^{-1}\left(\frac{R_{\mathrm{xzy},2,1}}{\sqrt{R_{\mathrm{xzy},2,2}^{2}+R_{\mathrm{xzy},2,3}^{2}}}\right)\\ \; & =\tan^{-1}\left(\frac{R_{2,1}}{\sqrt{R_{2,2}^{2}+R_{2,3}^{2}}}\right) \end{array}$$

(56) $$\begin{array}{cc} \mathrm{Pitch}= & \frac{180}{\pi }\cdot \mathrm{atan}2\left(\sin(\mathrm{TabAng}+q1+q2+th3)/2\right.\\ \; & \left.-\sin(q1-\mathrm{TabAng}+q2+th3)/2,\right.\\ \; & \left.\cos(\mathrm{TabAng}+q1+q2+th3)/2\right.\\ \; & \left.+\cos(q1-\mathrm{TabAng}+q2+th3)/2\right) \end{array}$$

(57) $$\begin{array}{cc} \mathrm{Yaw}= & \frac{180}{\pi }\cdot \mathrm{atan}2\left(-\cos(q1+q2+th3)\cdot \sin(\mathrm{Rot}),\right.\\ \; & \left.\cos(q1+q2+th3)\cdot \cos(\mathrm{Rot})\right) \end{array}$$

(58) $$\begin{array}{cc} \mathrm{Roll}= & \frac{180}{\pi }\cdot \mathrm{atan}2\left(\sin(q1+q2+th3),\right.\\ \; & \left.\sqrt{{\left(\frac{\cos(TabAng+q1+q2+th3)}{2}+\frac{\cos(q1-TabAng+q2+th3)}{2}\right)}^{2}}\right.\\ \; & \left.+{\left(\frac{\sin(TabAng+q1+q2+th3)}{2}-\frac{\sin(q1-TabAng+q2+th3)}{2}\right)}^{2}\right) \end{array}$$

## 5. Materials and Methods

### 5.1. Hardware Components

The patient positioning system (PPS) is built using state-of-the-art hardware components to ensure high precision and reliability. The primary components include the following:

- Kollmorgen Servo Motors: These motors provide high torque and precision, enabling smooth and accurate motion for the PPS across all axes.
- Renishaw Encoders: High-resolution 26-bit BiSS-C encoders (models RESA30USA200B and RA26BAA200B50F) are integrated into the system to deliver real-time feedback and enhance the accuracy of the positioning system.
- ACS Motion Controllers and Drivers: The system employs ACS controllers and drivers to manage the servo motors and process encoder data. These controllers enable synchronized multi-axis motion control, critical for the PPS’s six degrees of freedom (6-DOF) operation.

### 5.2. Control Software: SPiiPlus MMI Application Studio

The SPiiPlus MMI Application Studio, developed by ACS Motion Control, serves as the primary interface and development environment for the PPS. This software is used to perform the following tasks:

- System Setup: Configuring servo motors, encoders, and controllers to ensure seamless integration of hardware components.
- Axis Calibration and Tuning: Implementing precise axis calibration and tuning the dual-loop feedback system for optimal performance.
- Real-Time Monitoring: Providing a robust platform for real-time monitoring of system parameters, including motor positions, encoder feedback, and velocity profiles.
- Diagnostics and Debugging: Enabling detailed diagnostics for troubleshooting and optimizing the system’s performance.
- Forward Kinematics Implementation: Programming and deploying the FK model to compute the exact position of the patient bed during normal operation and emergency interruptions.

The SPiiPlus MMI Application Studio acts as a central hub, facilitating the efficient development and management of the PPS. It provides tools such as multi-channel scopes and wizards for axis configuration, enhancing the accuracy and reliability of the system.

### 5.3. Data Acquisition and Analysis

To evaluate the system’s performance during interruptions, real-time data are gathered from the ACS Motion Controllers using the SPiiPlus MMI Application Studio. The data include the following:

- Motor positions and velocities obtained from primary encoders.
- Load positions tracked by secondary encoders.
- System behavior during simulated interruption scenarios.

These data are exported and analyzed using MATLAB, where they are used to perform the following tasks:

- Visualize system responses to different interruption scenarios.
- Apply forward kinematics algorithms to estimate the system’s position during and after interruptions.
- Compare the FK-derived position with encoder feedback to evaluate accuracy and reliability.

### 5.4. Testing Forward Kinematics During Interruptions

The primary goal of this study is to verify the FK model’s ability to accurately determine the patient bed’s position during emergency scenarios, such as power loss or emergency stops. The steps include the following:

1. Simulate various interruption scenarios using the SPiiPlus MMI Application Studio.
2. Gather real-time encoder feedback during the interruptions.
3. Plot and analyze the data in MATLAB to assess the system’s recovery trajectory.
4. Validate the FK model by comparing its positional estimates with actual encoder data.

## 6. Results

The patient positioning system (PPS) was evaluated under three simulated interruption scenarios: power loss, emergency stop, and communication failure. These scenarios were tested during linear motion along the X-axis, Y-axis, and during 3D motion incorporating angular adjustments (Pitch, Yaw, Roll). The results emphasize the system’s ability to recover accurately from interruptions while maintaining precision and stability.

### 6.1. System Response During X-Axis Motion

**Figure 18:**
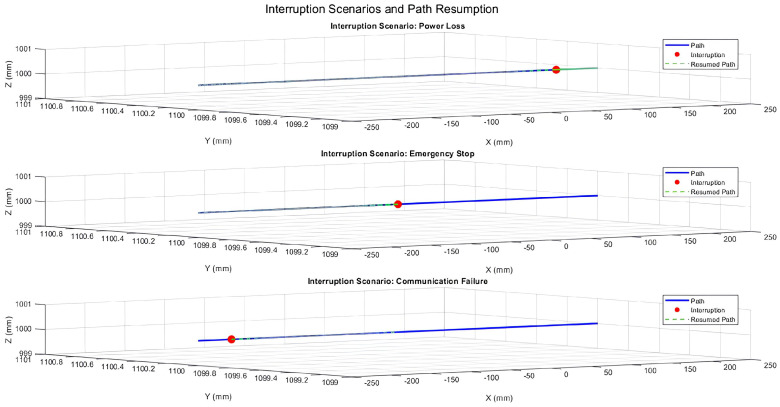
Three-dimensional trajectory illustrating interruption scenarios during X-axis motion with fixed Y and Z positions.

Figure 18 illustrates the PPS’s response during X-axis motion with the Y and Z positions fixed at 1100 mm and 1000 mm, respectively. The interruptions—a power loss at 20% of the trajectory, emergency stop at 50%, and communication failure at 80%—temporarily disrupted the motion. However, the system promptly resumed its planned trajectory after each interruption, maintaining sub-millimeter positional accuracy.

### 6.2. System Response During Y-Axis Motion

**Figure 19:**
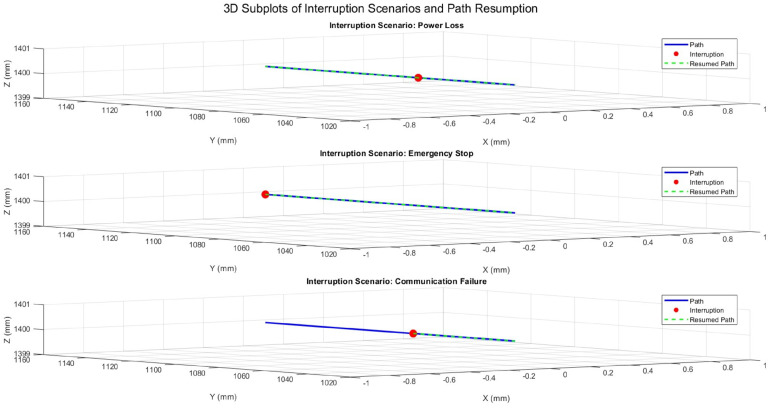
Three-dimensional trajectory illustrating interruption scenarios during Y-axis motion with fixed X and Z positions.

The system’s behavior during Y-axis motion with fixed X and Z positions is shown in Figure 19. The same interruption scenarios were simulated, and the resumed paths consistently aligned with the planned trajectory. This demonstrates the system’s ability to recover reliably, ensuring precision after disruptions.

### 6.3. System Response During 3D Motion with Angular Adjustments

**Figure 20:**
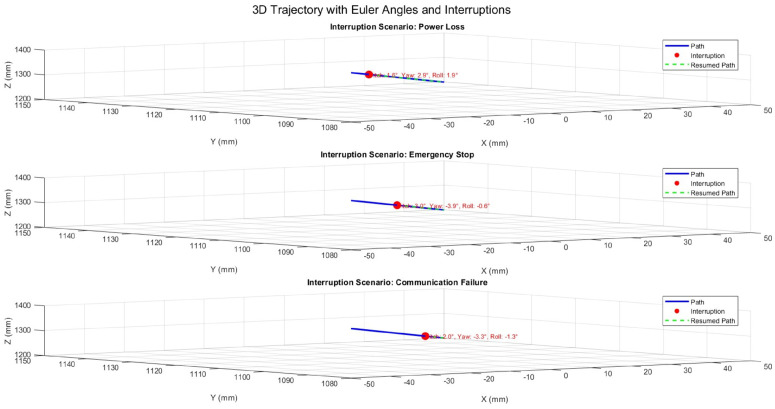
Three-dimensional trajectory illustrating interruption scenarios with Euler angles (Pitch, Yaw, Roll).

Figure 20 presents the system’s response during 3D motion, incorporating angular adjustments (Pitch, Yaw, Roll). The recorded angles at the interruption points reflect temporary deviations, but the system quickly regained angular stability. The maximum angular deviation observed was $0.1^{\circ }$, demonstrating effective recovery while maintaining stability.

### 6.4. Performance Metrics Summary

The system’s performance across all scenarios was quantified using key metrics, as summarized in Table 2. Positional accuracy was evaluated based on the deviation from the planned trajectory, while angular stability was assessed using deviations in Pitch, Yaw, and Roll. Recovery time was measured as the duration required to resume the planned trajectory after an interruption.

These results confirm the PPS’s ability to maintain high precision and stability under interruption scenarios. The system achieved a mean positional error of 0.05 mm, with a maximum deviation of 0.1 mm, and angular deviations remained under $0.1^{\circ }$ with a mean of $0.03^{\circ }$. Recovery times averaged 2.0 s, ensuring minimal delay in operation. The combination of rapid recovery, positional accuracy, and angular stability demonstrates the robustness of the PPS, making it highly suitable for radiosurgery applications.

## 7. Discussion

The results validate the system’s robustness in managing operational interruptions, demonstrating its ability to maintain precision and reliability under various scenarios. The dual-loop encoder feedback system played a pivotal role in ensuring accurate position and angular data retention, enabling precise trajectory resumption after power loss, emergency stops, and communication failures. Safe halting mechanisms effectively prevented positional drift during pauses, while secondary encoder feedback facilitated uninterrupted recovery, even under partial communication loss. These findings confirm the system’s capability for sub-millimeter positional accuracy and angular stability, making it highly suitable for high-precision applications in clinical environments.

In comparison with existing patient positioning systems (PPS), our robotic system demonstrates notable advantages. Commercial systems such as the 6-DoF Robotic Couch by gKteso GmbH and the Hexapod solutions by PI (Physik Instrumente) offer precision and reliability, with reported accuracies of up to 0.5 mm [19,20]. However, these systems are limited by their narrower travel ranges and reduced adaptability during treatment. In contrast, our system achieves a superior accuracy of 0.1 mm and features an extended travel range, enabling greater flexibility in handling complex treatment geometries. Furthermore, while systems developed by Siemens and Samsung focus on load capacity and integration with imaging technologies, they lack dynamic positioning adjustments during treatment [17,18]. By incorporating dual-loop feedback and forward kinematics (FK) for real-time trajectory adjustments, our system overcomes these limitations and establishes a new benchmark for adaptability and precision.

The effectiveness of our approach is further supported by existing research. Studies by Lan et al. [12] and Kim et al. [13] emphasize the importance of integrating primary and secondary encoder feedback to enhance system accuracy and reliability. Our implementation aligns with these findings, showcasing how redundancy in feedback mechanisms can mitigate the impact of communication failures and improve recovery performance. Additionally, Johnson and Patel (2022) highlight that dual-loop control architectures significantly outperform single-loop systems in maintaining alignment and stability [15]. Nguyen and Garcia (2021) further underscore the importance of robust safety mechanisms to improve patient outcomes [16], a consideration that our system addresses through its safe halting and recovery protocols [29].

Despite these strengths, certain limitations warrant further investigation. The system’s reliance on high-resolution encoder feedback introduces potential sensitivity to noise, which may require the development of advanced filtering algorithms to maintain consistent performance. Moreover, while the system performed reliably under controlled laboratory conditions, additional validation in dynamic clinical environments is essential to evaluate its robustness under real-world scenarios. This includes testing with variable patient anatomies, weights, and treatment trajectories. Finally, while the current design is optimized for intracranial procedures, its modular nature offers opportunities for adaptation to broader applications, such as spinal or thoracic treatments, through tailored hardware and software configurations.

Clinically, the implications of this work are significant. By maintaining sub-millimeter accuracy during interruptions, the system minimizes radiation exposure to healthy tissues and enhances treatment efficacy. The rapid recovery mechanisms reduce treatment delays, contributing to improved operational efficiency and patient comfort. These features position the system as a valuable advancement in robotic radiosurgery, addressing key challenges in precision, safety, and reliability. With future enhancements to algorithms, noise mitigation, and broader clinical validations, the system holds promise for further advancing the capabilities of medical robotics in high-stakes clinical settings beyond radiosurgery.

## 8. Conclusions

This study validates the performance and reliability of a robotic patient positioning system for radiosurgery, demonstrating its ability to maintain precision and recover seamlessly from operational interruptions. The system achieved sub-millimeter positional accuracy during and after interruptions, with robust recovery mechanisms ensuring the precise resumption of the trajectory following power loss, emergency stops, and communication failures. The integration of forward kinematics (FK) with real-time encoder feedback enabled reliable position and orientation tracking, both during normal operations and under interruption scenarios.

When compared to other systems currently in use, the proposed solution offers distinct advantages in terms of accuracy, stability, and recovery performance. By combining a 0.1 mm absolute accuracy with a wide travel range and advanced control architecture, the system addresses the critical limitations of existing robotic solutions. This capability ensures enhanced patient safety, improved clinical outcomes, and minimized treatment delays, making it a reliable and competitive solution for high-stakes clinical settings.

Future work will build upon these findings by expanding the system’s applications beyond intracranial procedures. While the current validation focused on brain tumor treatments, we aim to extend the system’s functionality to address lung tumors. This application presents unique challenges due to respiratory motion, requiring the integration of real-time tracking and advanced imaging techniques for dynamic tumor localization. Enhancing the system’s algorithms to process real-time imaging data and synchronize with the patient’s breathing cycle will be a key focus. Such developments will further validate the system’s robustness and versatility, ensuring it meets the demanding requirements of treating complex and mobile targets in radiosurgery.

These advancements will position the proposed system at the forefront of precision robotics for medical applications, offering broader utility across a wide range of clinical scenarios while setting new benchmarks in safety, accuracy, and reliability.

## 9. Patents

The patient positioning system (PPS) is presently undergoing the patenting process, holding the application number PCT/US2019/048205.

## Figures and Tables

**Table 2:** Performance metrics for system stability and recovery.

Metric	Mean	Standard Deviation	Maximum
Positional Error (mm)	0.05	0.02	0.1
Angular Deviation (°)	0.03	0.02	0.1
Recovery Time (s)	2.0	0.2	2.2

## Data Availability

The data presented in this study are available on request from the corresponding author.
